# BUILDING BRIDGES TO FUTURE LEADERS: A CAREER PATHWAYS WEBINAR

**DOI:** 10.1093/geroni/igac059.301

**Published:** 2022-12-20

**Authors:** Meghan McDarby

**Affiliations:** Memorial Sloan Kettering Cancer Center, New York, New York, United States

## Abstract

Educational webinars can improve attitudes toward careers in aging. We developed a 6-session webinar series about careers in geropsychology, detailing unique career pathways (e.g., academic, private practice, VA). Our evaluation of the series included trainees from graduate programs and postdoctoral fellowships. Participants rated their attitudes toward each career pathway at pre- and post-session. At post-session, participants reported significantly increased interest in careers in aging (t(83) = 4.72, p < .00). They also reported significantly increased understanding of the type of training required for careers in aging (t(77) = 8.10, p < .00). Importantly, at post-session, participants reported stronger beliefs that they could be successful in a career in aging (t(84) = 3.86, p < .00) and that they would have good work-life balance (t(87) = 9.34, p < .00). Results suggest that a webinar series may increase student interest in and understanding of unique career pathways in geropsychology.

